# Digital Therapeutics–Based Cardio-Oncology Rehabilitation for Lung Cancer Survivors: Randomized Controlled Trial

**DOI:** 10.2196/60115

**Published:** 2025-02-25

**Authors:** Guangqi Li, Xueyan Zhou, Junyue Deng, Jiao Wang, Ping Ai, Jingyuan Zeng, Xuelei Ma, Hu Liao

**Affiliations:** 1 Department of Biotherapy, Cancer Center and State Key Laboratory of Biotherapy West China Hospital Sichuan University Chengdu China; 2 West China School of Medicine Sichuan University Chengdu China; 3 Department of Biotherapy, State Key Laboratory of Biotherapy, Frontiers Science Center for Disease-related Molecular Network West China Hospital Sichuan University Chengdu China; 4 Key Laboratory of Bio-Resource and Eco-Environment of Ministry of Education College of Life Sciences Sichuan University Chengdu China; 5 Department of Rehabilitation Medicine West China Hospital Sichuan University Chengdu China; 6 Department of Head and Neck Oncology, Cancer Center West China Hospital Sichuan University Chengdu China; 7 Department of Thoracic Surgery West China Hospital Sichuan University Chengdu China

**Keywords:** cardio-oncology rehabilitation, digital therapeutics, telerehabilitation, non-small cell lung cancer, exercise prescription, cardiology, oncology, rehabilitation, cardiorespiratory fitness, cardiopulmonary, cancer, physical activity, digital health, digital technology, randomized controlled trial, wearable, app, quality of life, survivor

## Abstract

**Background:**

Lung cancer ranks as the leading cause of cancer-related deaths. For lung cancer survivors, cardiopulmonary fitness is a strong independent predictor of survival, while surgical interventions impact both cardiovascular and pulmonary function. Home-based cardiac telerehabilitation through wearable devices and mobile apps is a substitution for traditional, center-based rehabilitation with equal efficacy and a higher completion rate. However, it has not been widely used in clinical practice.

**Objective:**

The objective of this study was to broaden the use of digital health care in the cardiopulmonary rehabilitation of lung cancer survivors and to assess its impact on cardiopulmonary fitness and quality of life (QOL).

**Methods:**

Early-stage nonsmall cell lung cancer survivors aged 18-70 years were included. All the participants received surgery 1-2 months before enrollment and did not require further antitumor therapy. Participants were randomly assigned to receive cardiac telerehabilitation or usual care for 5 months. Artificial intelligence–driven exercise prescription with a video guide and real-time heart rate (HR) monitoring was generated based on cardiopulmonary exercise testing. Aerobic exercise combining elastic band–based resistance exercises were recommended with a frequency of 3-5 d/wk and a duration of 90-150 min/wk. The effective exercise duration was recorded when patients’ HR reached the target zone (HR_resting_ + [HR_max_ – HR_resting_] × [≈40%-60%]), representing the duration under the target intensity. The prescription used a gradual progression in duration and action intensity based on the exercise data and feedback. Outcome measurements included cardiopulmonary fitness; lung function; cardiac function; tumor marker; safety; compliance; and scales assessing symptoms, psychology, sleep, fatigue, and QOL.

**Results:**

A total of 40 (85%) out of 47 patients finished the trial. The average prescription compliance rate of patients in the telerehabilitation group reached 101.2%, with an average exercise duration of 151.4 min/wk and an average effective exercise duration of 92.3 min/wk. The cardiac telerehabilitation was associated with higher improvement of maximal oxygen uptake peak (3.66, SD 3.23 mL/Kg/min vs 1.09, SD 3.23 mL/Kg/min; *P=.*02) and global health status or QOL (16.25, SD 23.02 vs 1.04, SD 13.90; *P=.*03) compared with usual care. Better alleviation of affective interference (–0.88, SD 1.50 vs 0.21, SD 1.22; *P=.*048), fatigue (–8.89, SD 15.96 vs 1.39, SD 12.09; *P=.*02), anxiety (–0.31, SD 0.44 vs –0.05, SD 0.29; *P=.*048), and daytime dysfunction (–0.55, SD 0.69 vs 0.00, SD 0.52; *P=.*02) was also observed in the telerehabilitation group. No exercise-related adverse events were identified during the intervention period.

**Conclusions:**

The 5-month, digital therapeutics–based telerehabilitation improved cardiorespiratory fitness in lung cancer survivors with good compliance and safety. Patients receiving telerehabilitation also reported improved QOL with reduced levels of fatigue, anxiety, and daytime dysfunction.

**Trial Registration:**

Chinese Clinical Trial Registry ChiCTR2200064000; https://www.chictr.org.cn/showproj.html?proj=180594

## Introduction

Cardio-oncology is a specialized field dedicated to studying and managing cardiovascular health in cancer therapy. It has emerged in response to an increasing awareness of the intricate interplay between the 2 disciplines [1]. Globally, the incidence of lung cancer is 64.1 cases per 100,000 individuals in men and 50.3 cases per 100,000 individuals in women. Lung cancer ranks as the second most commonly diagnosed type of cancer overall and the leading cause of cancer-related deaths. For lung cancer survivors, cardiopulmonary fitness is of paramount importance due to its multifaceted impact on overall health, quality of life (QOL), and long-term outcomes [2-7]. Parameters such as maximal oxygen uptake peak (VO_2_ peak), predicted postoperative forced expiratory volume in 1 second, and predicted postoperative diffusing capacity of the lungs for carbon monoxide are commonly used to assess surgical risks and are correlated with the prognosis of lung cancer survivors [2,7]. As the primary therapeutic intervention for localized nonsmall cell lung cancer (NSCLC), surgical interventions impact both cardiovascular and pulmonary function [8-13] and increase cardiovascular disease (CVD) risk [14-16]. Therefore, incorporating cardio-oncology rehabilitation into postoperative care for NSCLC survivors should be considered an indispensable component [17]. In addition, previous studies have also reported that exercise could reduce psychological symptoms [18], sleep dysfunction [19], and fatigue [20] and improve QOL [21] in lung cancer survivors.

The effectiveness and safety of traditional medical monitoring under center-based cardiac rehabilitation (CBCR) have been thoroughly validated. Numerous studies indicate that CBCR significantly reduces the incidence of secondary cardiovascular events, lowers hospital readmission rates, and decreases mortality among patients with CVDs [22-25]. Cardiac rehabilitation in China has rapidly expanded with multilevel hospitals gradually offering rehabilitation services. However, the significant investment in terms of manpower, equipment, and facilities, as well as time costs and transportation limitations, have constrained the participation rate, completion rate, and economic efficiency of CBCR. The scarcity of specialized cardiac rehabilitation personnel is a primary factor contributing to low adherence in overpopulated countries like China [26]. In the United States, less than 20% of individuals requiring cardiac rehabilitation participate in such programs [27,28]. The low adherence of cancer survivors to exercise rehabilitation and the need for individualized prescriptions further hinder the progress of CBCR. Home-based cardiac rehabilitation (HBCR) is a viable alternative to the traditional CBCR model. Numerous clinical studies have demonstrated that the effectiveness and safety of HBCR are comparable to CBCR, particularly in cases of stable coronary heart disease, chronic heart failure, acute myocardial infarction, and patients undergoing vascular reconstruction [29,30]. Notably, participation and completion rates in HBCR surpass those in CBCR [29,30]. In a meta-review, 63% of the 25 virtual reality–based exercise rehabilitation programs for cancer-related dysfunctions reached a patient-reported compliance rate of more than 85% [31], although data on cardiopulmonary fitness in NSCLC survivors are still insufficient. An additional advantage lies in the cost-effectiveness of HBCR, making it a more economical option. The European Society of Cardiology, the American Association of Cardiovascular and Pulmonary Rehabilitation, and the Chinese Association of Rehabilitation Medicine have each provided recommendations and guidance on HBCR in the years 2016, 2019, and 2021 [32-34].

Digital health, often associated with the use of information technology, serves to promote health and deliver health care services. This encompasses a range of platforms such as websites, mobile text messages, smartphone apps, and wearable devices embedded with various sensors [35]. For cardio-oncology telerehabilitation, wearable devices using Bluetooth or Wi-Fi technology facilitate remote monitoring of metrics like heart rate (HR), respiratory rate, electrocardiogram, physical activity, and other health indicators [36]. These technologies can enhance patient engagement and adherence in HBCR, strengthen communication between health care providers (HCPs) and patients, assist in managing CVD risk factors, and reduce health care expenses [33,37-39]. Digital therapeutics (DTx)–based rehabilitation has been shown to improve cardiopulmonary fitness, particularly among patients with cardiac disease and those infected with COVID-19 [40-42]. Cardio-oncology telerehabilitation has also been studied to relieve treatment toxicity and prevent CVD [43-45]. Despite this, there are limited studies specifically addressing its effects on patients with lung cancer after surgery. Recovery Plus Health is a software as medical device approved by the Chinese National Medical Products Administration for remote cardiac rehabilitation and prehabilitation. With the app for HCPs, they can (1) check, modify, or confirm the artificial intelligence (AI)–driven tailored exercise prescription and send it to patients; and (2) check the feedback information from patients and optimize the exercise prescription dynamically. With the app for patients, they can (1) do exercise rehabilitation following the tailored exercise prescription video guide and AI-driven instructions; (2) receive real-time HR monitoring with individualized safety alerts; and (3) provide exercise-specific feedback. This study aimed to evaluate the efficacy, safety, and compliance of a 5-month cardio-oncology rehabilitation program based on the Recovery Plus Health app and wearable devices for NSCLC survivors after surgery.

## Methods

### Trial Design and Randomization

This is a single-blind, prospective, randomized controlled trial (RCT) to determine the effectiveness, safety, and compliance of a DTx-based cardiopulmonary rehabilitation program. Random numbers were generated before the start of the enrollment by a researcher unrelated to this trial. The main researcher enrolled participants and assigned them to interventions according to the random number and the entry order. The grouping was blinded to patient identity. Outcomes were analyzed blinded to the study group. The reporting of this trial adheres to the CONSORT (Consolidated Standards of Reporting Trials) 2010 statement (Multimedia Appendix 1).

### Ethical Considerations

This trial was approved by the Research Ethics Board of the West China Hospital of Sichuan University (2022-1317) and registered at the Chinese Clinical Trial Register (ChiCTR2200064000). The study was conducted in accordance with the Declaration of Helsinki. Written informed consent was obtained from all participating patients and they were allowed to withdraw the consent anytime during the study for any reason. Data were collected at West China Hospital. Patients’ data are anonymized. Each patient received a subsidy of RMB 800 (≈US $110).

### Inclusion and Exclusion Criteria

The patients were recruited from West China Hospital. Patients who met all of the following criteria were included:

1. Early-stage NSCLC survivors after surgery who did not require radiotherapy or chemotherapy
2. Patients who underwent surgery 1-2 months before enrollment
3. Patients aged 18-70 years
4. Patients who own and are proficient in the use of a smartphone

Patients who met any of the following criteria were excluded:

1. Patients who had engaged in organized aerobic training within the preceding 3 months
2. Patients who had severe orthopedic or cardiopulmonary disease (including severe COVID-19 infection)
3. Patients with cognitive dysfunction or psychological disorders resulting in an inability to understand the content of the study

### Sample Size Calculation

According to previous research from Messaggi-Sartor et al [46] on the improvement of VO_2_ peak in postoperative exercise intervention for NSCLC, the sample size in this study was estimated to detect a difference of 2.13 mL/Kg/min in VO_2_ peak, with a SD of 2.3 mL/Kg/min, a type I error rate α of 0.05, a power of 0.90 (1 – β), and an intraclass correlation coefficient of 0.10 for the multicenter design. It was determined that 45 participants would be required for an independent 2-sample *t* test comparison. Considering a 10% attrition rate, the intended total sample size was 50 (25 in the intervention group and 25 in the control group). PASS (version 15.0; NCSS) was used for sample size calculation.

### Intervention

**Figure 1:**
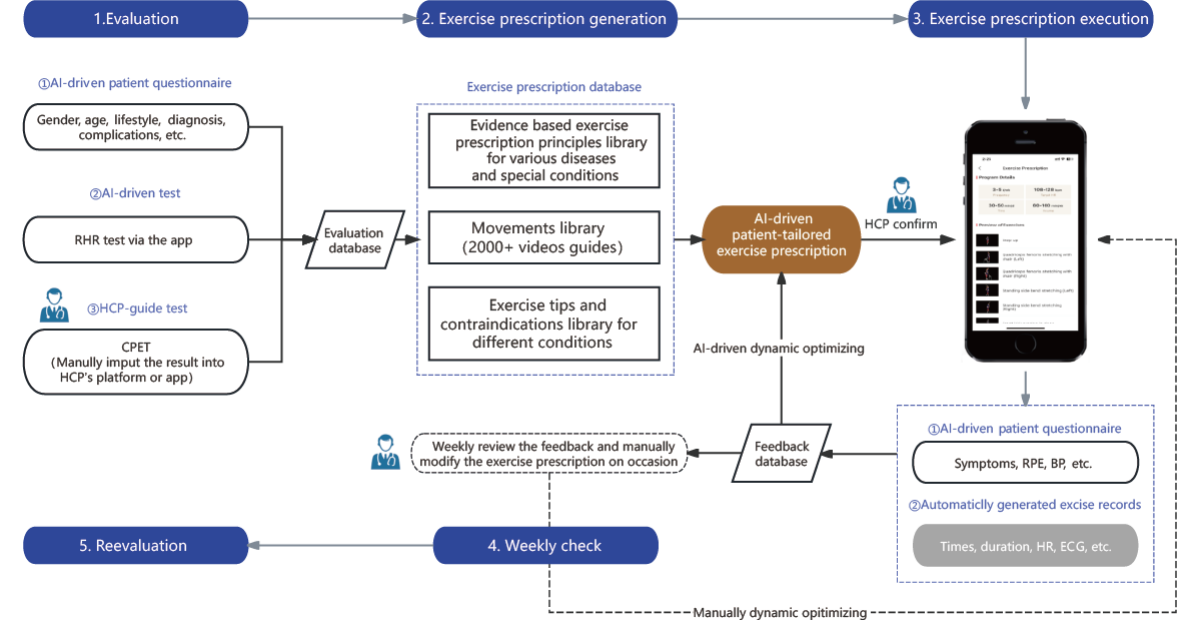
Diagram of the AI-driven exercise prescription system. AI: artificial intelligence; BP: blood pressure; CPET: cardiopulmonary exercise test; ECG: electrocardiogram; HCP: health care provider; HR: heart rate; RHR: resting heart rate; RPE: rating of perceived exertion.

Participants in the usual care group received routine postoperative rehabilitation guidance including condition monitoring, medication guidance, dietary guidance, oral exercise guidance, and psychological care. Besides the usual care, participants in the intervention group received exercise prescriptions with a video guide and real-time monitoring using the Recovery Plus Health app (Recovery Plus USA Inc), a software as medical device approved by the National Medical Products Administration for remote cardiac rehab and prehab. They were instructed to download the app for patients (Recovery Plus Inc; Multimedia Appendix 2), which was wirelessly connected to a chest-worn HR band (Recovery Plus Inc) to monitor exercise frequency, intensity, time, volume, and progression. These participants were shown how to operate the app and the equipment and were required to adhere to the prescribed exercises available on the app, wear the HR band during exercise, and provide feedback about the specific exercise after each rehabilitation session. The exercise prescriptions were generated according to the result of the cardiopulmonary exercise testing (CPET) and patients’ previous exercise habits. The prescription principle was based on suggestions for patients with cancer in American College of Sports Medicine’s guidelines [47] and Expert Consensus on Exercise Rehabilitation for Chinese Cancer Patients Centered on Functional Impairments [48]. Generally, aerobic exercise combining elastic band-based resistance exercises was recommended with a frequency of 3-5 d/wk and a duration of 90-150 min/wk. For each participant, the prescription was dynamically changed and highly personalized. Patient-reported, disease-related symptoms were considered during action selection. The effective exercise duration was recorded when patients’ HR reached the target HR zone, representing the duration under the target intensity. The lower number of the target HR zone was calculated as HR_resting_ + (HR_max_ – HR_resting_) × 40%. The higher number of the target HR zone was calculated as HR_resting_ + (HR_max_ – HR_resting_) × 60%. The prescription used a gradual progression and automatic adjustment in duration and action intensity based on the exercise data and feedback. All the adjustments automatically generated by the system were approved by the physician. The HCP reviewed the exercise records and feedback information via the Recovery Plus Health app for HCPs (Multimedia Appendix 3) weekly and made necessary modifications to the exercise regimen, while the app automatically recorded compliance with the exercise prescription. Regardless of the group, all participants were instructed to discontinue exercising and promptly report to their clinician or exercise professional if any cardiac-related symptoms occurred. Moreover, HCPs were available 24/7 to receive and respond to alerts from the participants. The intervention began 1-2 months after the surgery. Figure 1 illustrates the operational diagram of the AI-driven exercise prescription system.

### Measurements

Outcome measurements included cardiopulmonary fitness (VO_2_ peak; primary outcome); lung function (forced expiratory volume in 1 second and diffusing capacity of the lungs for carbon monoxide); cardiac function (tricuspid annular plane systolic excursion and ejection fraction); cytokeratin 19 fragmentation antigen; safety; compliance (completion rate, exercise times per week, effective exercise duration, and prescription adherence rate); and scales assessing symptoms (MD Anderson Symptom Inventory for Patients With Lung Cancer [49,50]), psychology (Hospital Anxiety and Depression Scale [51]), sleep (Pittsburgh Sleep Quality Index [52]), fatigue (Multidimensional Fatigue Symptom Inventory—Short Form [53,54]), and QOL (European Organization for Research and Treatment of Cancer Quality of Life Questionnaire-Core 30 [55]). All the scales have been studied for validity and reliability. The assessment was conducted at baseline (1-2 months after surgery) and 5 months after the intervention, respectively. All the examinations were performed at West China Hospital and the scales were collected via the web. Exercise data including frequency, duration, HR, and subjective feedback were recorded by the app.

### Statistical Analysis

Descriptive statistics are presented as frequency distributions, means, and SD. Fisher exact test was used for unordered qualitative data. Independent 2-sample *t* tests were used for normally distributed continuous quantitative data with equal variance. The Mann-Whitney *U* test was used for ordered qualitative data and nonnormally distributed or heteroscedastic continuous quantitative data. The distribution of data was analyzed by the Shapiro-Wilk test. The equality of variances was tested using the Levene test. Statistical significance was established at a 2-tailed *P* value of less than .05. All analyses were conducted using an intention-to-treat approach.

## Results

### Participants and Compliance

**Figure 2:**
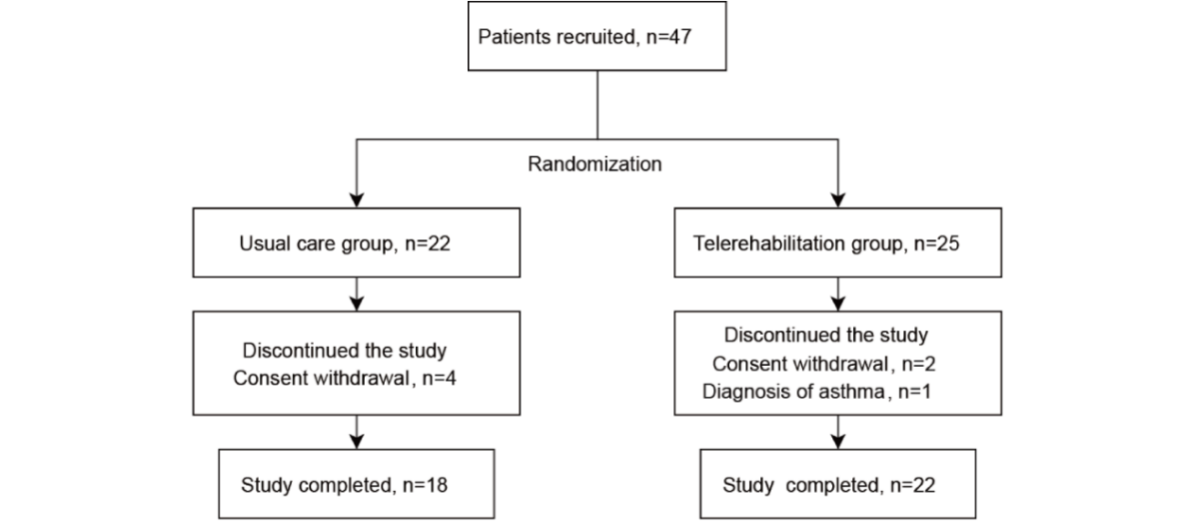
CONSORT (Consolidated Standards of Reporting Trials) study flow diagram.

From September 2022 to September 2023, a total of 47 patients in West China Hospital were enrolled in the study, with 40 (85%) individuals completing the trial (22 in the telerehabilitation group and 18 in the usual care group). Seven participants withdrew from the trial. One participant in the telerehabilitation group was diagnosed with asthma and the other 6 withdrew their consent because they were unable to come back to the hospital for examination on time due to living far away (Figure 2). The baseline data of participants who completed the study are presented in Table 1. The adherence rate of the telerehabilitation group was 88% (22/25). Patients in this group exercised 3.33 times a week on average, with an average exercise duration of 151.4 min/wk. The average effective exercise duration (reaching the prescribed target HR zone) was 92.25 min/wk. The mean prescription adherence rate (effective exercise duration to the lower limit of prescription duration) was 101.2%.

**Table 1:** Baseline data of participants completed the study.

Characteristics		Total (n=40)	Usual care (n=18)	Telerehabilitation (n=22)	*P* value	
Age (years), mean (SD)		51.33 (11.32)	53.50 (10.71)	49.50 (11.66)	.27	
**Sex, n (%)**						.25
	Male	9 (22.5)	6 (33.3)	3 (13.6)		
	Female	31 (77.5)	12 (66.7)	19 (86.4)		
**American Joint Committee on Cancer stage, n (%)**						.75
	IA1	16 (40)	7 (38.9)	9 (40.9)		
	IA2	13 (32.5)	7 (38.9)	6 (27.3)		
	IA3	7 (17.5)	3 (16.7)	4 (18.2)		
	IB	4 (10)	1 (5.6)	3 (13.6)		
**Pathology, n (%)**						>.99
	Adenocarcinoma	39 (97.5)	18 (100)	21 (95.5)		
	Squamous-cell carcinoma	1 (2.5)	0 (0)	1 (4.5)		
VO_2_ peak^a^ (mL/Kg/min), mean (SD)		19.43 (4.51)	19.98 (4.18)	18.97 (4.82)	.49	
MET^b^, mean (SD)		6.12 (1.33)	6.29 (1.27)	5.99 (1.4)	.53	
FEV_1_^c^ (L), mean (SD)		2.2 (0.49)	2.16 (0.5)	2.23 (0.5)	.69	
DLCO^d^ (mmol/min/kPa), mean (SD)		20.44 (4.3)	20.7 (4.92)	20.23 (3.88)	.75	
TAPSE^e^ (mm), mean (SD)		20.86 (2.97)	21.5 (2.94)	20.32 (2.96)	.25	
EF^f^, mean (SD)		67.63 (4.92)	67 (4.07)	68.14 (5.56)	.47	
CYFRA21^g^ (ng/mL), mean (SD)		1.85 (0.7)	1.9 (0.78)	1.81 (0.65)	.72	

^a^VO_2_ peak: maximal oxygen uptake peak.

^b^MET: metabolic equivalent of task.

^c^FEV_1_: forced expiratory volume in 1 second.

^d^DLCO: diffusing capacity of the lungs for carbon monoxide.

^e^TAPSE: tricuspid annular plane systolic excursion.

^f^EF: ejection fraction.

^g^CYFRA21: cytokeratin 19 fragmentation antigen.

### Efficacy and Safety

Four participants did not receive a CPET after the intervention and another 4 participants did not complete the scales on time. A total of 36 participants accepted both baseline and postintervention CPETs, with 20 individuals in the telerehabilitation group and 16 in the usual care group. Additionally, 36 participants responded to the baseline and postintervention scales, with 20 individuals in the telerehabilitation group and 16 in the usual care group. The cardiac telerehabilitation was associated with higher improvement of VO_2_ peak (3.66, SD 3.23 mL/Kg/min vs 1.09, SD 3.23 mL/Kg/min; *P=.*02) and global health status or QOL (16.25, SD 23.02 vs 1.04, SD 13.90; *P=.*03) compared with usual care. Better alleviation of affective interference (relationships with others, enjoyment of life, and emotions; (–0.88, SD 1.50 vs 0.21, SD 1.22; *P=.*048), fatigue (–8.89, SD 15.96 vs 1.39, SD 12.09; *P=.*02), anxiety (–0.31, SD 0.44 vs –0.05, SD 0.29; *P=.*048), and daytime dysfunction (–0.55, SD 0.69 vs 0.00, SD 0.52; *P=.*02) were also observed in the telerehabilitation group (Table 2). No significant differences in other efficacy indicators were observed between the 2 groups (all *P*>.05). No exercise-related adverse events were identified during the intervention period.

**Table 2:** Effectiveness outcomes changing for both groups.

Outcomes					Usual care (n=16), mean (SD)	Telerehabilitation (n=20), mean (SD)		*P* value
VO_2_ peak^a^ (mL/Kg/min)					1.09 (3.23)	3.66 (3.23)		.02
FEV_1_^b^ (L)					0.20 (0.17)	0.25 (0.23)		.53
DLCO^c^ (mmol/min/kPa)					1.74 (2.10)	0.84 (1.87)		.23
TAPSE^d^ (mm)					–2.00 (4.40)	0.00 (3.24)		.27
EF^e^ (%)					–0.60 (4.90)	–2.33 (5.86)		.37
CYFRA21^f^ (ng/mL)					0.13 (1.04)	0.44 (0.67)		.42
**MDASI-LC^g^**								
	Mean core symptom severity				0.05 (1.12)	–0.70 (1.53)		.11
	Mean module symptom severity				–0.26 (1.47)	–0.63 (1.16)		.40
	Mean total symptom severity				–0.21 (2.29)	–1.33 (2.35)		.16
	Mean interference				–0.06 (1.44)	–0.79 (1.64)		.08
	Mean activity interference				–0.33 (1.84)	–0.70 (2.07)		.17
	Mean affective interference				0.21 (1.22)	–0.88 (1.50)		.048
**EORTC^h^ QLQ-C30^i^**								
	**Functional scales**							
		Physical functioning		–1.67 (15.3)		6.00 (8.06)	.11	
		Role functioning		9.38 (21.05)		6.67 (13.68)	.58	
		Emotional functioning		–0.52 (16.52)		4.58 (13.10)	.24	
		Cognitive functioning		–6.25 (13.44)		0.00 (10.81)	.13	
		Social functioning		3.13 (20.38)		11.67 (18.81)	.32	
	**Symptom scales or items**							
			Fatigue	1.39 (12.09)		–8.89 (15.96)	.02	
			Nausea and vomiting	1.04 (11.33)		–1.67 (11.97)	.53	
			Pain	–6.25 (20.07)		–8.33 (17.52)	.97	
			Dyspnea	–6.25 (18.13)		–6.67 (25.59)	.97	
			Insomnia	8.33 (22.77)		0.00 (28.61)	.31	
			Appetite loss	–6.25 (18.13)		0.00 (15.29)	.26	
			Constipation	6.25 (25.00)		0.00 (15.29)	.31	
			Diarrhea	2.08 (14.75)		1.67 (7.45)	.88	
			Financial difficulties	0.00 (17.21)		–1.67 (22.88)	.60	
			Global health status or quality of life	1.04 (13.90)		16.25 (23.02)	.03	
**HADS^j^**								
	Anxiety				–0.05 (0.29)	–0.31 (0.44)		.048
	Depression				0.01 (0.37)	–0.22 (0.38)		.07
**PSQI^k^**								
	Subjective sleep quality				0.06 (0.44)	–0.10 (0.64)		.47
	Sleep latency				–0.38 (1.41)	–0.20 (1.15)		.92
	Sleep duration				–0.44 (1.36)	0.05 (0.76)		.22
	Habitual sleep efficiency				0.19 (0.98)	–0.12 (0.86)		.33
	Sleep disturbances				–0.25 (0.86)	–0.20 (0.83)		.92
	Use of sleeping medication				0.00 (0.63)	0.25 (0.79)		.71
	Daytime dysfunction				0.00 (0.52)	–0.55 (0.69)		.02
	Global score				–0.81 (3.64)	–0.94 (3.19)		.57
**MSFI-SF^l^**								
	General Fatigue				–0.05 (0.49)	–0.10 (0.89)		.51
	Physical Fatigue				–0.13 (0.63)	–0.06 (0.65)		.76
	Emotional Fatigue				–0.20 (0.48)	–0.29 (0.84)		.65
	Mental Fatigue				0.11 (0.43)	0.00 (0.52)		.49
	Vigor				–0.20 (1.04)	0.10 (0.98)		.41
	Total Score				–0.06 (2.29)	–0.55 (2.66)		.57

^a^VO_2_ peak: maximal oxygen uptake peak.

^b^FEV_1_: forced expiratory volume in 1 second.

^c^DLCO: diffusing capacity of the lungs for carbon monoxide.

^d^TAPSE: tricuspid annular plane systolic excursion.

^e^EF: ejection fraction.

^f^CYFRA21: cytokeratin 19 fragmentation antigen.

^g^MDASI-LC: MD Anderson Symptom Inventory for Patients With Lung Cancer.

^h^EORTC: European Organization for Research and Treatment of Cancer.

^i^QLQ-C30: Quality of Life Questionnaire-Core 30.

^j^HADS: Hospital Anxiety and Depression Scale.

^k^PSQI: Pittsburgh Sleep Quality Index.

^l^MSFI-SF: Multidimensional Fatigue Symptom Inventory—Short Form.

## Discussion

### Strengths and Clinical Implications

This RCT shows the effects of DTx-based telerehabilitation for NSCLC survivors. Both cardiopulmonary fitness and QOL were significantly improved by telerehabilitation compared with usual care. Previous research has demonstrated that exercise-based rehabilitation can enhance cardiopulmonary fitness across a diverse range of disease conditions. A similar RCT in early-stage NSCLC survivors has shown that 8-week aerobic exercise combining high-intensity respiratory muscle training improved VO_2_ peak (2.13 mL/Kg/min). However, in this CBCR program, only 69% (11/16) participants persisted in the exercise group with a compliance (session completion rate) of 80%, and no significant QOL improvement was observed [46]. Similar low adherence has been reported in many rehabilitation studies for NSCLC [56,57], especially in advanced NSCLC survivors after surgery [58,59]. As for the impact of exercise interventions on the QOL, conflicting findings have been reported in lung cancer survivors [60]. A meta-analysis involving 34 RCTs demonstrated that unsupervised HBCR did not improve QOL in cancer survivors compared with supervised programs [61]. Patients in unsupervised programs often fail to reach the recommended exercise dosage, which is the main reason for the observed absence of QOL improvement. Maintaining high adherence to exercise routines is a crucial approach to ensure the benefits [60]. Integrating wearable devices into HBCR presents a flexible solution that overcomes the limitations of traditional CBCR, offering patients a more adaptable and convenient way to access rehabilitation programs. In this study, 88% (22/25) of the patients in the telerehabilitation group completed the trial with an impressive average prescription compliance rate of 101.2%, indicating a strong engagement and adherence to the prescribed exercise regimen. DTx enables patients to actively participate in their health management by delivering ongoing feedback and encouragement, motivating them to maintain regular physical activity.

Exercise type affects the outcome of rehabilitation. Aerobic training is particularly effective in improving VO_2_ peak and associated cardiopulmonary variables while resistance training primarily focuses on enhancing overall muscular health. An RCT reported that aerobic training rather than resistance training improved VO_2_ peak in lung cancer survivors with poor cardiorespiratory fitness [62]. However, a combination of aerobic exercise and resistance exercise can enhance various health outcomes commonly affected by cancer [63]. Another factor that affects the outcome of rehabilitation is the intensity. High-intensity exercise, characterized by a metabolic equivalent of task greater than 6, is defined as an activity that induces sweating and elevates heart and respiratory rates. It has been shown to decrease cancer progression and enhance survival rates in breast, colon, and prostate cancers, with emerging evidence suggesting similar benefits in lung cancer [64]. The exercise prescription in this program combined aerobic and resistance exercises and displayed real-time HR on the guidance interface to help patients maintain an effective exercise intensity.

In addition to good compliance and effectiveness, the following 2 features of this system make it promising for clinical applications. First, the tag-based action recommendation system allows for a high degree of individualization. This is reflected not only in action selection based on patient symptoms and duration settings according to previous exercise habits but also in the ability to adjust action intensity and duration dynamically based on exercise data and feedback. Second, this system enables clinicians to manage a large number of patients simultaneously via the web, viewing patient exercise data and feedback intuitively, thus achieving high management efficiency. These characteristics are difficult to achieve with existing CBCR systems.

### Limitations

This study has certain limitations that warrant consideration. First, the relatively short duration of the intervention, which was limited to 5 months, did not allow us to observe long-term outcomes such as prognosis. Implementing the intervention through an app introduces a learning curve for patients. The novelty of digital therapeutics can also be a limitation. Therefore, a longer follow-up period is needed to understand the sustained effects on disease progression and participants’ awareness, knowledge, attitudes, intentions to change, help-seeking behaviors, and behavior change, providing a fuller picture of the long-term benefits of HBCR. In addition, a rehabilitation regimen that encompasses a broader time frame, including the perioperative period, could potentially yield greater benefits. Second, the focus of this study was on exercise prescription, which, while a central aspect of cardiac rehabilitation, is only one part of a complex intervention. Comprehensive cardiac rehabilitation programs should also include nutrition guidance, psychological support, medication management, smoking cessation efforts, patient education, and diligent monitoring of key health indicators. Incorporating these multifaceted components could significantly enhance the program’s effectiveness and the overall wellness of lung cancer survivors. Third, this study is centered on patients with early-stage, postoperative NSCLC due to their extended survival expectancy, which correlates with a heightened risk of CVDs in the future. They can tolerate most of the exercise prescriptions and intensities, which positions them as the ideal candidates for telerehabilitation following lung surgery. However, for patients with advanced NSCLC, additional treatments such as radiotherapy and chemotherapy can lead to more significant side effects and cardiopulmonary damage. It is essential to conduct further studies to investigate telerehabilitation modalities and their effectiveness for these patients.

### Conclusions

This study aimed to expand the application of digital health care in cardiopulmonary rehabilitation after lung cancer surgery. The 5-month, DTx-based telerehabilitation improved cardiorespiratory fitness in lung cancer survivors with good compliance and safety. Patients receiving telerehabilitation also reported improved QOL with reduced levels of fatigue, anxiety, and daytime dysfunction. The findings indicate that incorporating DTx-based cardiac rehabilitation into postoperative care may benefit early-stage NSCLC survivors. More work should be done to address the long-term impact on prognosis.

## Appendix

Multimedia Appendix 1 CONSORT (Consolidated Standards of Reporting Trials) checklist.

Multimedia Appendix 2 App for patients.

Multimedia Appendix 3 App for health care providers.

## Abbreviations

AI: artificial intelligence
CBCR: center-based cardiac rehabilitation
CPET: cardiopulmonary exercise test
CONSORT: Consolidated Standards of Reporting Trials
CVD: cardiovascular disease
DTx: digital therapeutics
HBCR: home-based cardiac rehabilitation
HCP: health care provider
HR: heart rate
NSCLC: nonsmall cell lung cancer
QOL: quality of life
RCT: randomized controlled trial
VO2 peak: maximal oxygen uptake peak
