# Supplementary figures and images for: Digital Therapeutics–Based Cardio-Oncology Rehabilitation for Lung Cancer Survivors: Randomized Controlled Trial
Source: JMIR Mhealth Uhealth. 2025 Feb 25;13:e60115. doi: 10.2196/60115 (PMC11897676; doi:10.2196/60115)

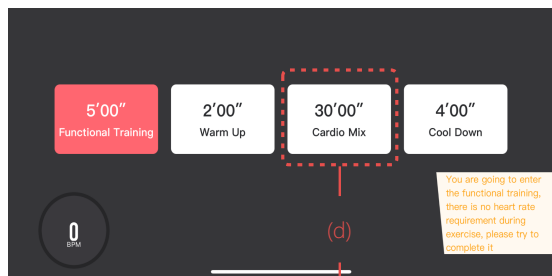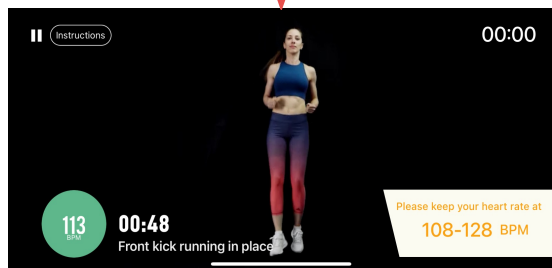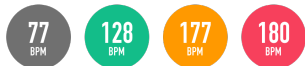

Visual & Auditory Alert according to Ex Principles

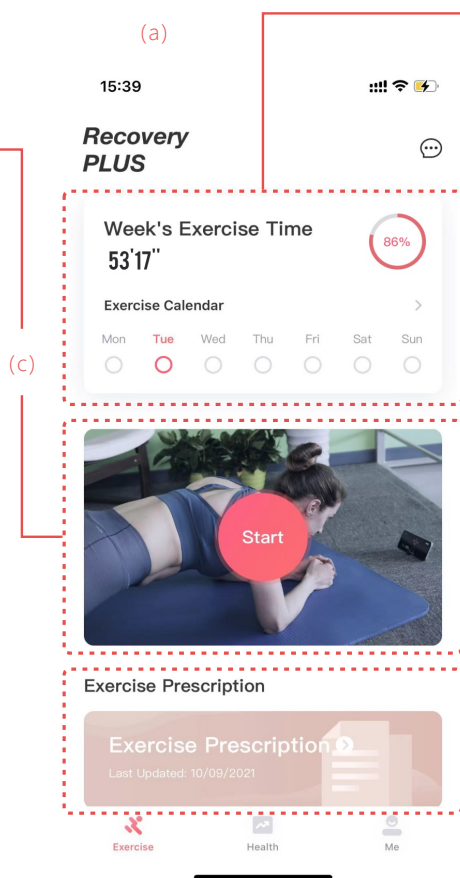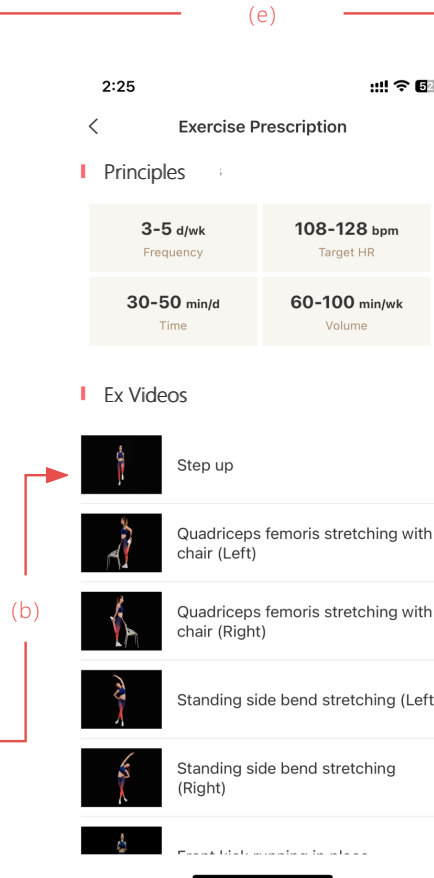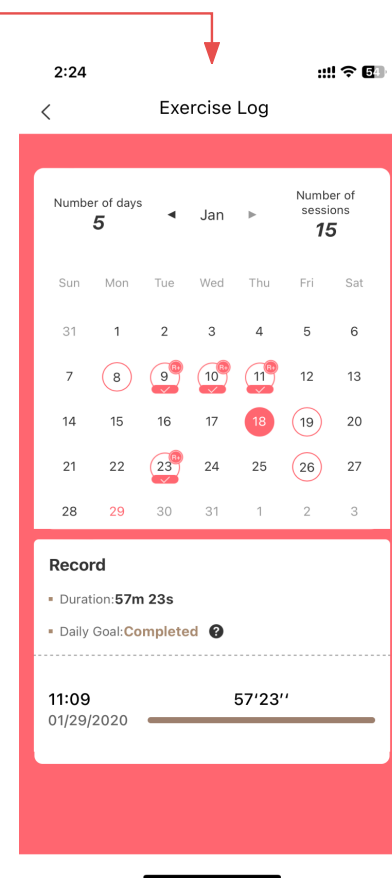

Supplement: Multimedia Appendix 2 [file mhealth_v13i1e60115_app2.pdf]
