# Supplementary material for: Digital Therapeutics–Based Cardio-Oncology Rehabilitation for Lung Cancer Survivors: Randomized Controlled Trial
Source: JMIR Mhealth Uhealth. 2025 Feb 25;13:e60115. doi: 10.2196/60115 (PMC11897676; doi:10.2196/60115)
Supplement: Multimedia Appendix 3 [file mhealth_v13i1e60115_app3.pdf]

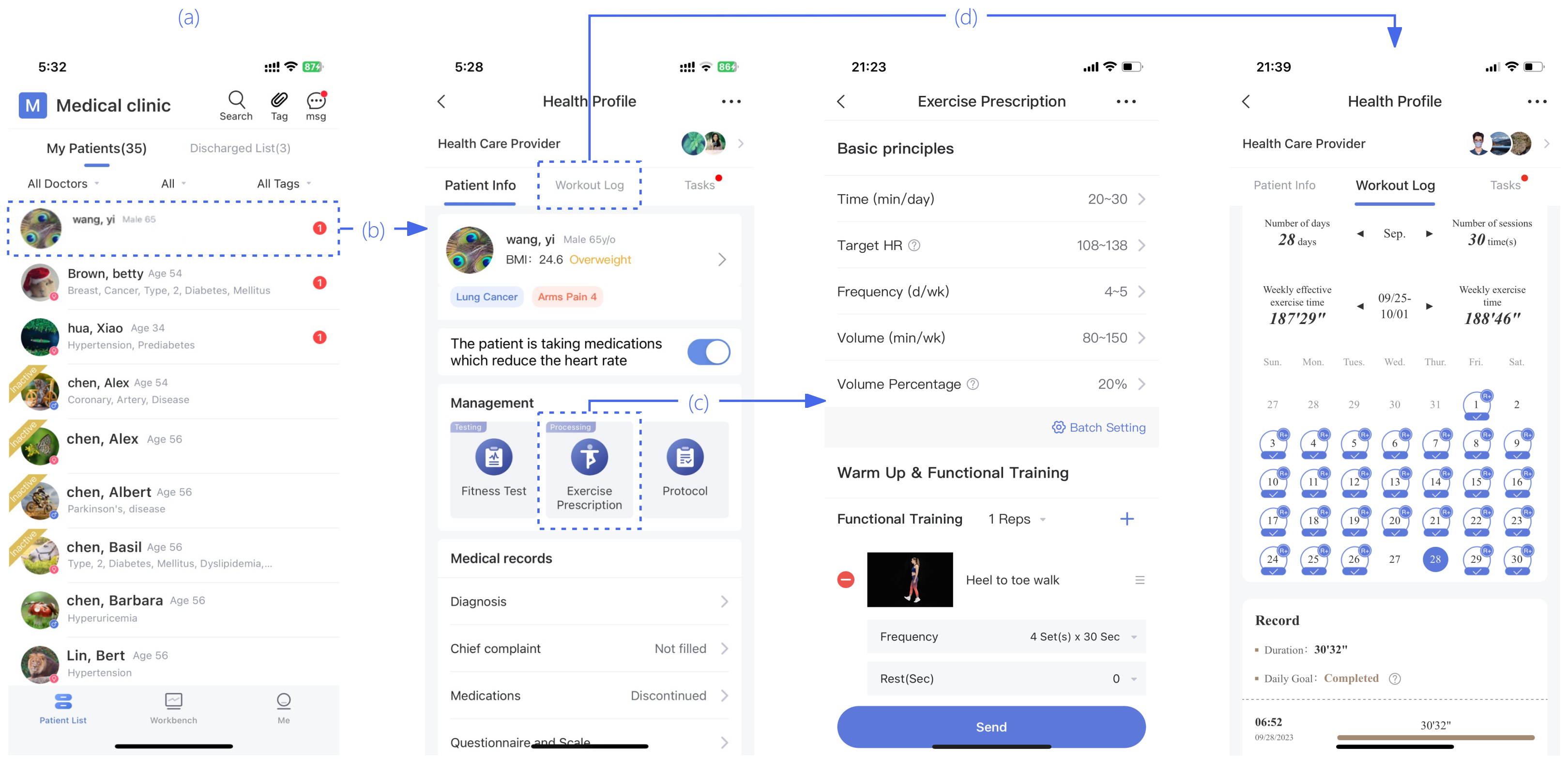
(a)
(b)
(c)
(d)
5:32
Medical clinic
Search
Tag
msg
My Patients(35)
Discharged List(3)
All Doctors
All
All Tags
wang, yi Male 65
Brown, betty Age 54
Breast, Cancer, Type, 2, Diabetes, Mellitus
hua, Xiao Age 34
Hypertension, Prediabetes
chen, Alex Age 54
Coronary, Artery, Disease
chen, Alex Age 56
chen, Albert Age 56
Parkinson's, disease
chen, Basil Age 56
Type, 2, Diabetes, Mellitus, Dyslipidemia,...
chen, Barbara Age 56
Hyperuricemia
Lin, Bert Age 56
Hypertension
Inactive
Patient List
Workbench
Me
5:28
Health Profile
Health Care Provider
Patient Info
Workout Log
Tasks
wang, yi Male 65y/o
BMI: 24.6 Overweight
Lung Cancer
Arms Pain 4
The patient is taking medications which reduce the heart rate
Management
Testing
Processing
Fitness Test
Exercise Prescription
Protocol
Medical records
Diagnosis
Chief complaint Not filled
Medications Discontinued
Questionnaire and Scale
21:23
Exercise Prescription
Basic principles
Time (min/day) 20~30
Target HR 108~138
Frequency (d/wk) 4~5
Volume (min/wk) 80~150
Volume Percentage 20%
Batch Setting
Warm Up & Functional Training
Functional Training 1 Reps
Heel to toe walk
Frequency 4 Set(s) x 30 Sec
Rest(Sec) 0
Send
21:39
Health Profile
Health Care Provider
Patient Info
Workout Log
Tasks
Number of days 28 days
Sep.
Number of sessions 30 time(s)
Weekly effective exercise time 187'29"
09/25-10/01
Weekly exercise time 188'46"
Sun. Mon. Tues. Wed. Thur. Fri. Sat.
Record
Duration: 30'32"
Daily Goal: Completed
06:52
09/28/2023
30'32"
